# Removal of *Pseudomonas fluorescens* biofilms from pilot-scale food processing equipment using ozone-assisted cleaning-in-place

**DOI:** 10.3389/fmicb.2023.1141907

**Published:** 2023-04-14

**Authors:** Goksel Tirpanci Sivri, Ahmed G. Abdelhamid, David R. Kasler, Ahmed E. Yousef

**Affiliations:** ^1^Department of Food Science and Technology, The Ohio State University, Columbus, OH, United States; ^2^Department of Food Engineering, Faculty of Agriculture, Tekirdağ Namık Kemal University, Tekirdağ, Türkiye; ^3^Botany and Microbiology Department, Faculty of Science, Benha University, Benha, Egypt; ^4^Department of Microbiology, The Ohio State University, Columbus, OH, United States

**Keywords:** biofilm, *Pseudomonas* spp., cleaning-in-place, ozone, dairy industry, food spoilage, sanitizers

## Abstract

Biofilm formation in food processing environment and within equipment increases the risk of product spoilage and contamination with pathogens. Cleaning-in-place (CIP) operations are useful in removing soils and in sanitizing processing equipment, including eliminating biofilms. However, CIP is a resource-intensive process, particularly in the usage of chemical detergents, heat, and sanitizers. The current study was initiated to investigate the feasibility of integrating ozone into CIP operations to facilitate the elimination of *Pseudomonas* biofilm, with the long-term goal of decreasing the dependance on conventional cleaning and sanitizing reagents. To investigate integrating ozone into CIP, a robust biofilm of *Pseudomonas fluorescens* was developed on a pilot-scale food processing equipment after 2 days of incubation in 10% skim milk (skim milk-water mixture, 1:9 v/v) under stagnant conditions, followed by additional 5 days of circulation while feeding 10% fresh skim milk. CIP was applied using water prerinse at 22–25°C, alkaline cleaning with 0.2% potassium hydroxide at 50°C, and a final water rinse. These CIP operations reduced planktonic cell populations below the detection method’s limit but did not fully remove *P. fluorescens* biofilm from either smooth or rough surfaces of the processing equipment. When the CIP process was followed by application of an aqueous ozone step (10 ppm for 10 min), the treatment reduced biofilm cell population, on smooth and rough surfaces, below the recovery method’s detection limit (0.9 and 1.4 log CFU/ 100 cm^2^, respectively). These findings demonstrate the utility of ozone-assisted CIP in eliminating microbial biofilms on processing equipment, but further research is needed to optimize the use of cleaning agents and the application of ozone.

## 1. Introduction

Control of psychrotrophic spoilage and pathogenic bacteria (e.g., *Pseudomonas* spp. and *Listeria monocytogenes*, respectively) in food processing environment has been difficult to achieve. Under suitable moisture and nutrient conditions, these bacteria can quickly develop biofilms on food processing equipment (Brooks and Flint, 2008). The resulting biofilm serves as a continuous source for repeated food product contamination, which largely impacts product’s shelf-life and safety. In fact, the role of biofilm in the food industry, particularly the dairy sector, has been studied extensively (Panebianco et al., 2022), and researchers found that bacteria can form biofilms at several points of the dairy processing operation and in many parts of the processing equipment (Marchand et al., 2012). Biofilms of spoilage microorganisms such as *Pseudomonas* spp. deemed as a challenging problem in dairy processing operations. Members of the genus *Pseudomonas* are ubiquitous and are frequently isolated from the dairy production equipment or dairy products. *Pseudomonas* spp. produce thermostable lipases and proteases which could persist after thermal treatment of milk and hence cause spoilage of the end products (Zhang et al., 2019) through production of undesirable flavors and odors (Reichler et al., 2021). Several *Pseudomonas* spp. are commonly isolated from dairy factories; these include *P. fluorescens*, *P. fragi*, *P. putida*, *P. entomophila*, and *P. aeruginosa* (Chiesa et al., 2014).

Cleaning-in-place (CIP) operations are designed to ensure the safety of processed food by minimizing product recontamination during processing (Seiberling, 1997). CIP systems remove deposited materials on interior surface of equipment without the need to open or dismantle the equipment and with little or no manual operation (Smithers, 2022). Conventional CIP operations vary in effectiveness to remove biofilm formed on food contact surfaces (Dufour et al., 2004). Such operation typically consists of these steps; (1) prerinsing with water, (2) alkali cleaning with or without subsequent acid cleaning and (3) sanitization. The sanitization step is designed to maintain sufficient hygienic condition for the equipment. This step commonly involves using biocides including chlorine, peracetic acids, iodophores, or quaternary ammonium compounds (Joseph et al., 2001). However, bacteria in the biofilm state can develop protection against the antimicrobial action of these biocidal agents (Srey et al., 2013; Wassenaar et al., 2015). Extensive use of these biocides may negatively impact human health and cause deterioration of processing equipment (Marino et al., 2018). As an alternative sanitizer, ozone has been exploited for many applications in the food industry, and it is approved for food treatment, storage, and processing (Food and Drug Administration, 2001). Ozone is characterized by its strong antimicrobial activity against many pathogenic and spoilage microorganisms and is recognized as an eco-friendly sanitizer because of its minimal environmental impact (Pascual et al., 2007; Marino et al., 2018; Masotti et al., 2019; Bigi et al., 2021). Ozone inactivates microorganisms by damaging their cytoplasmic membranes and other cellular components through a variety of mechanisms including oxidation of unsaturated lipids, protein, and nucleic acids (Khadre et al., 2001). Ozone also can prevent the initial attachment of microbial cells to surfaces and suppress the formation of biofilms by damaging the extracellular matrix of participating cells (Panebianco et al., 2022).

Formation of biofilm by *Pseudomonas* spp. in processing environment is a considerable problem in the food industry, which can lead to product spoilage and disease outbreaks (Srey et al., 2013). Systematic studies about the generation of *Pseudomonas* biofilms in an industrial setting and the use of ozone against such biofilms are lacking. Hence, the current study was initiated to (1) investigate the ability of *P. fluorescens* to form a robust biofilm on pilot-scale food processing equipment when a skim milk (an example of a dairy product) is supplied as a nutrient source, and (2) assess the efficacy of a biofilm control technology in which ozone is used as a sanitizer within CIP decontamination process.

## 2. Materials and methods

### 2.1. Ozone-assisted cleaning-in-place system

**Figure 1:**
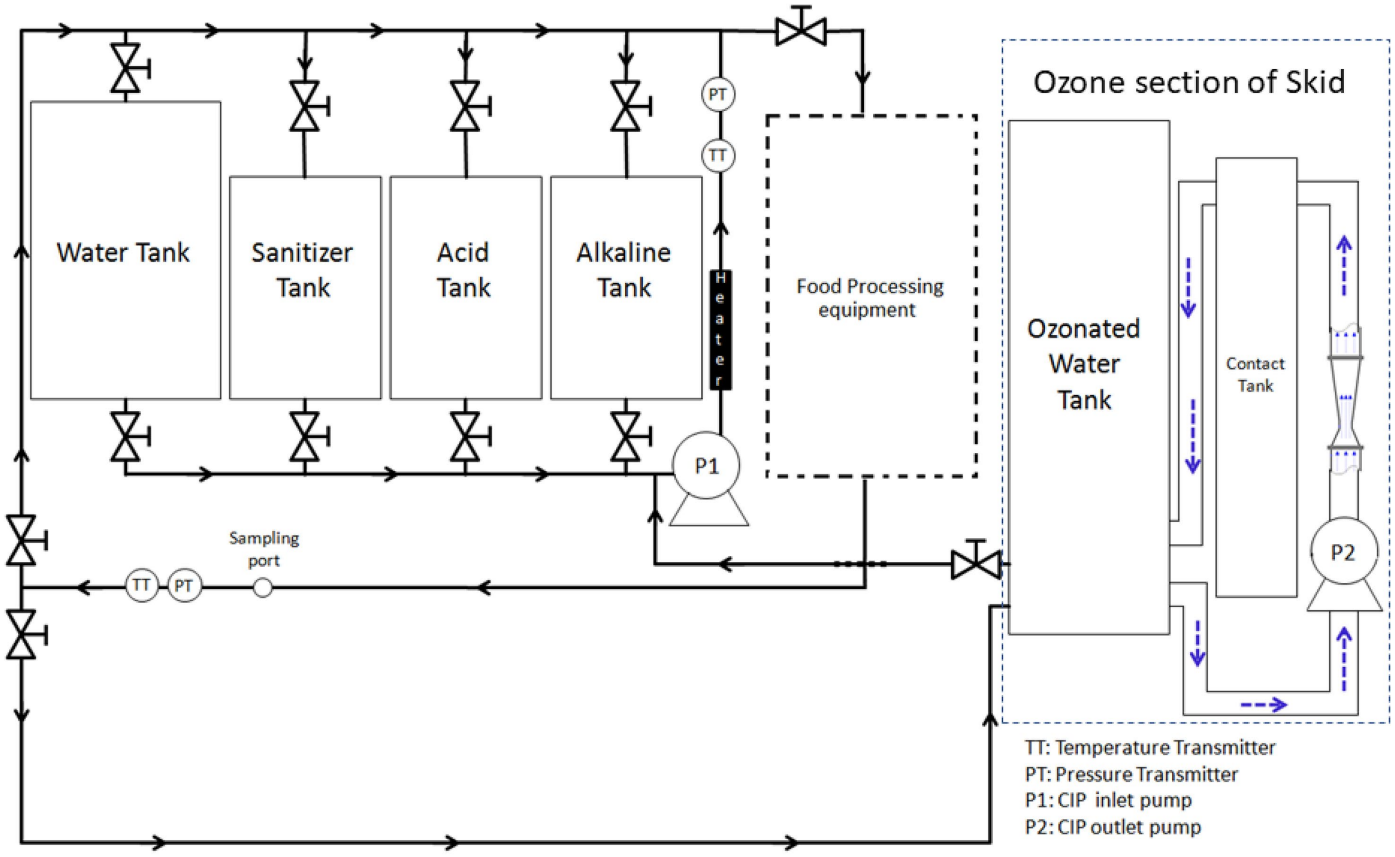
Representation of pilot-scale cleaning-in-place system integrated with ozone generator and coupled with a pilot-scale food processing equipment.

Ozone-assisted CIP system (Figure 1) was custom-made for this study through a cooperative effort between researchers at The Ohio State University and an ozone equipment manufacturer (Del Ozone, San Luis Obispo, CA). The system (Figure 1) is comprised of a 132-L tank for holding rinse water, three 57-L tanks (for holding alkaline solution, acid solution, and sanitizer), a heat exchanger (Model no: STFT-6000-240; TruHeat, Richmond Hill, ON, Canada), a pump, and meters for measuring turbulent flow, temperature, pH, and conductivity. A centrifugal pump (Gould centrifugal pump, model NPE 1ST1F5B6, Seneca Falls, NY) was used to circulate CIP fluid to the pilot-scale food processing equipment and was regulated by GS AC Drive (Automation Direct, Cumming, GA). Monitoring and data acquisition was performed with two flow meters (Blue-White Industries F-1000, Huntington Beach, CA), two thermocouples (Omega, Stamford, CT), a pH meter (Jenco Instr., San Diego, CA) with a pH probe, and a conductivity meter (Emerson/Rosemount Analytical Model 1,056 Dual Input Analyzer, Shakopee, MN) with two conductivity probes (Emerson). These probes were placed in both inlet and return lines of the CIP system, in relevance to the processing equipment. CIP equipment was set up in a way that by opening the proper valves, the CIP pump could pull fluid from any single tank (water, alkaline, acid, or sanitizer) and either direct the flow to the processing equipment through a spray ball; the flow then would run through all the processing lines and return the water back to the starting tank, or simply recirculate the water through the heater and back to the tank to control temperature. The ozone sanitation system (Del Ozone AGW 4045), having a built-in ozone generator and controller (Del Ozone Genesis CD-45GV), was integrated with the CIP system. The ozone part of the skid had its own circulation loop with a second pump (Model no: NPE 1ST1F1B4, Gould Pumps) to continuously generate and maintain the ozonated water as it was being used. The ozone system was setup so that the CIP pump could pull water from the main ozone water tank to sanitize the processing equipment and then water was collected in the rinse tank to be reused for next CIP treatments; therefore, the amount of wastewater could be minimized.

### 2.2. Pilot-scale food processing equipment

**Figure 2:**
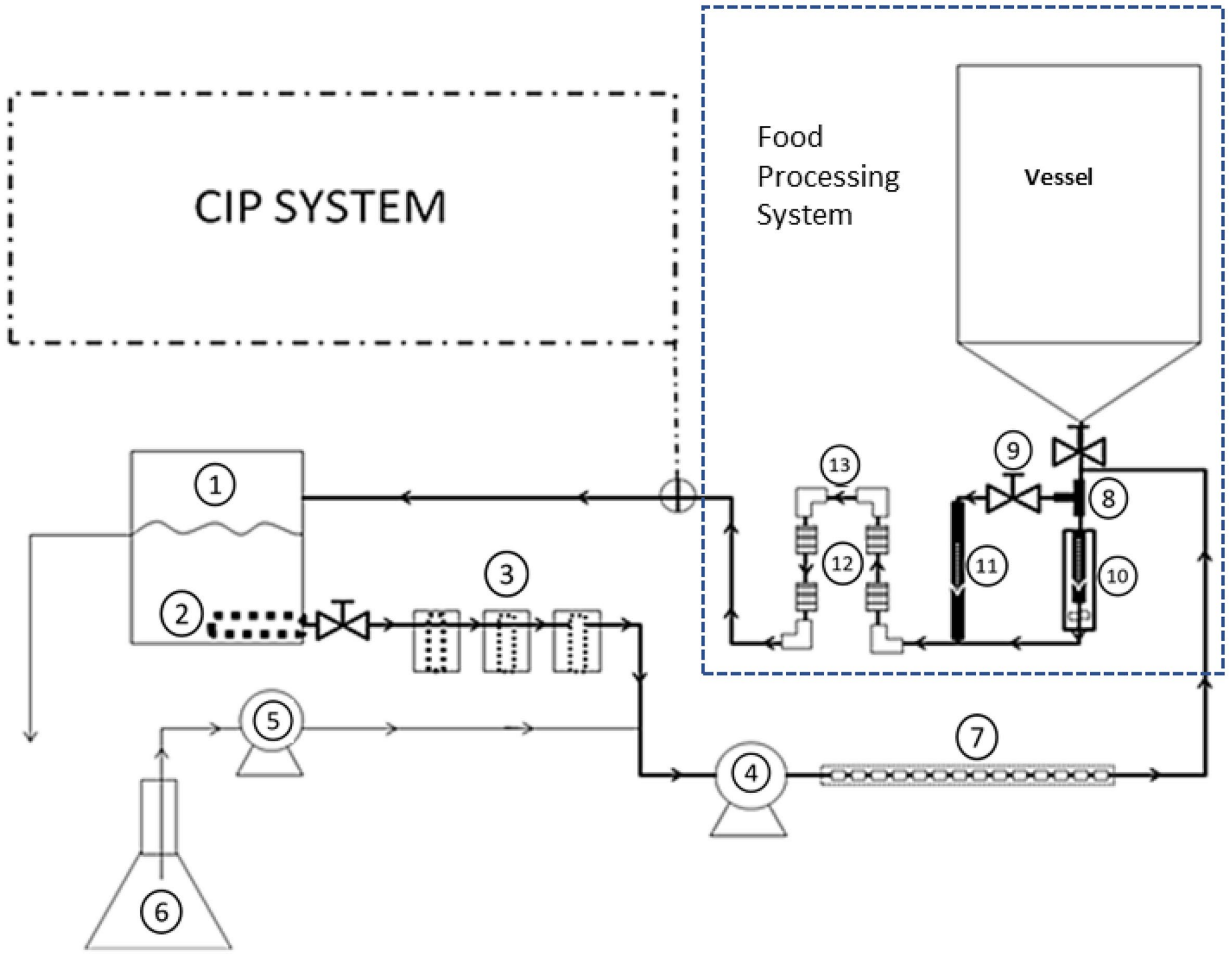
Representation of experimental set-up that includes the fouling system used to contaminate the pilot-scale processing equipment and to develop dairy biofilm on stainless steel surfaces. The fouling equipment consisted of the following parts, as numbered in the figure: (1) 19-L high density polyethylene container, (2) internal filter with 4.8 mm perforations, (3) three external filters on the line with mesh size of 20, 40, and 40, (4) peristaltic pump for circulation, (5) peristaltic pump for feeding line, (6) 5-L feeding flask, and (7) small stainless-steel tubes inserted into neoprene tubing. Components of the processing equipment included: stainless steel T-shaped pipe (8), by-pass valve (9), filler (10), by-pass line (11), four of rough surface pipe segments (12), and four smooth surface 90°-elbows (13).

The CIP system was attached to a pilot-scale setup that simulates food processing equipment, which was made of stainless steel, grade 304 (Figure 2). The processing equipment was constructed to be easily connectable to the CIP system during cleaning. The processing equipment consisted of: (a) 190-L tank (vessel), (b) filler valve placed inside a larger pipe, (c) pipe tee, (d) by-pass line, (e) four small pipe segments (2.54 cm internal diameter, 5.08 cm long and internal surface area of 40.5 cm^2^), which were artificially made with rough surface using a grit silicon carbide ball cylinder hone (Brush Research manufacturing, CA), (f) four of 90°-elbows (2.54 cm internal diameter and internal surface area 117.0 cm^2^), which had smooth surfaces, (g) return pump (Gould Pump, Model NPE 1ST1C5E6), and (h) 7.6 M (25 ft) of 2.54-cm diameter pipe.

### 2.3. Fouling system

The desired experimental biofilm needed to be robust and to simulate what naturally would take place when cleaning of a food processing equipment is neglected or inefficiently completed. To make the study manageable and the experiments repeatable, the biofilm was generated during a 7 day process. To generate this biofilm, a fouling system was developed that consisted of a 19-L high density polyethylene container that holds up to 7.6 L of inoculated growth medium, which was then circulated slowly through the system. The equipment had a four-step filter system that allowed larger particles to be removed from the fluid stream before it reached the pump. The first filter was placed inside the container as a suction filter (4.8 mm perforations; McMaster-Carr, Columbus, OH). The other three filters (with mesh sizes of 20, 40, 40; McMaster-Carr) were external to the container and were designed to be removed and cleaned during the contamination run without total system disassembly or draining. After being pulled through the filters, the fluid enters a peristaltic pump (Masterflex Model no: 7553–70; Masterflex head model no: 7015; Barrington, IL) that was designated as “circulation pump” which transfers the contaminated fluid through the target processing equipment parts (Figure 2). A second 5-L reservoir containing sterilized media connected to a second peristaltic pump (Masterflex Model no: 7521–50; head model no: 7016; Masterflex) was tied into the input line of the fouling system to slowly add fresh media to keep the organism growing over the multiple–day incubation. The fouling system contained multiple small stainless-steel tubes (each was 6.35 mm internal diameter and 9.05 mm long and had internal surface area of 3.78 cm^2^) inserted into norprene tubing (06402–15; Cole Parmer, Vernon Hills, IL); the tubes were used to examine robustness of biofilm formation during contamination with *P. fluorescens*.

### 2.4. Preparation of *Pseudomonas fluorescens* cultures

*Pseudomonas fluorescens* ATCC 25289 was obtained from the culture collection of the Department of Microbiology at The Ohio State University (Columbus, OH). The frozen stock culture of the bacterium was subcultured in trypticase soy broth (TSB; Becton Dickinson & Co., Sparks, MD) and incubated at 30°C for 48 h in a shaker incubator (New Brunswick Scientific Co. Edison, NJ) with mild agitation. The *P. fluorescens* culture was spread onto tryptic soy agar (TSA; Becton Dickinson & Co.) and incubated at ambient temperature (22–25°C) for 48 h before subculturing in TSB prior to use in experiments (Meyer and Abdallah, 1978).

### 2.5. *Pseudomonas fluorescens* biofilm development

To induce biofilm formation, the fouling system (7.6-L capacity) was operated as follows. Skim milk (Kroger Co., Cincinnati, OH), 760 mL, was diluted with distilled water to a total volume of 7.6 L, and this 10% milk preparation was then autoclaved at 121°C for 30 min; this will be referred to as “biofilm medium.” After the biofilm medium was cooled to ambient temperature (22–25°C), it was inoculated with 38 mL (0.5% v/v) of *P. fluorescens* ATCC 25289 culture. The inoculated biofilm medium was circulated through the loop for 30 min, left stagnant for 2 days, then circulation was resumed at a speed of 1.5–2.0 L/h for 5 days. While inoculated medium was circulating in the main fouling loop, the 5-L flask was filled with fresh biofilm medium, and the contents were pumped with the second peristaltic pump at a speed of 125 mL/h to add new nutrients for *P. fluorescens* to continue growth (Figure 2).

During this biofilm development, biofilm samples (i.e., the fouling loop stainless-steel tubes) were taken out at these points: before the circulation start (i.e., after the 2-day stagnant incubation), and every day during the circulation for 5 days. Every biofilm sample taken consisted of 2 tubes, one for *P. fluorescens* enumeration and the other for examination by scanning electron microscope as described in later section.

### 2.6. Use of the ozone-assisted CIP for removal of *Pseudomonas fluorescens* biofilm from food processing equipment

All elements of the processing equipment were washed with detergent solution and rinsed, before running experiments. These elements, except the product tank, were sterilized by autoclaving at 121°C for 30 min. For development of biofilm on the equipment prior to CIP implementation, the fouling fluid was added between the tank and the outlet piping and allowed to flow through the filler, bypass, the 5.08-cm small pipe segments, and elbows, mentioned earlier, before return to the fouling loop. Preliminary testing showed that the processing tank was easy to clean and, thus, the tank outlet piping was the main target of the study. Therefore, the parts that were subject to fouling were the T-shaped pipe (8), by-pass valve (9), filler (10), by-pass line (11), four small pipe segments with the rough surfaces (12), and the four 90°-elbows with the smooth surfaces (13). The inoculated biofilm medium was circulated first through the loop for 30 min, left stagnant for 2 days, then circulation was resumed for 5 days, as described earlier. After a robust *P. fluorescens* biofilm developed, CIP process was implemented by applying the following steps sequentially: (1) pre-rinsing, (2) alkaline cleaning with post-rinsing, and (3) ozone-based sanitization. The pipe segments (Figure 2, component 12) and elbows (Figure 2, component 13) were the parts used to assess CIP step effectiveness in removing *P. fluorescens* biofilm. After each CIP step, a segment and elbow were removed, replaced fresh sterile parts, and their inner surfaces were swabbed to evaluate biofilm removal.

#### 2.6.1. Pre-rinsing

Once the biofilm-formation process was completed, the contaminated processing equipment was connected to the CIP system, followed by removing the fouling system. Pre-rising was performed by using 35-μm filtered tap water at 22–25°C temperature. The rinsing water was delivered from the water tank *via* the CIP inlet pump at a single pass with a speed of 56.7 L/min to ensure a turbulent flow; the velocity of the fluid was 1.87 m/s. The rinsing time was determined as 1 min which ensured the removal of all milk soils.

#### 2.6.2. Alkaline cleaning

After pre-rinsing with water, the alkaline solution was prepared by mixing 35-μm filtered tap water and alkaline detergent (CIP 100; Steris, Mentor, OH) to achieve a concentration of 0.2%. The solution was heated to 50°C, by circulating through the in-line heater. The temperature was managed by a temperature controller (Omron E5C2, Allied Electronics & Automation, Worthington, OH). When the temperature became stable, the processing equipment was filled with the alkaline solution, which was then circulated at a speed of 56.7 L/min for 2 min. The temperature (50 ± 2°C) and flow speed (1.87 m/s) were kept under control during the cleaning process.

#### 2.6.3. Post-rinse

Post-rinse eliminated alkali traces from the system being cleaned and it also cooled the system to make it ready for ozone sanitization. The 35-μm filtered tap water was delivered from water tank, which was the same tank used for water pre-rinse. The rinsing water was introduced to the system and conductivity of the solution in both inlet and outlet line was determined. When the desired water conductivity (300–320 μsc at 22–25°C) was reached, the rinsing time was continued for an extra minute before rinsing was completed and the pumps were shut down. Water flow was kept stable as 56.7 L/min.

#### 2.6.4. Ozone sanitization

Aqueous ozone solution was used as a sanitizer at ambient (22–25°C) temperature. Air was used as inlet gas in the ozone generator since it had an integrated oxygen concentrator system. Concentrated oxygen was converted into ozone *via* corona discharge method, and it was mixed with water by the help of a venturi device. The aqueous ozone solution was stored in ozone-water tank (Figure 1) and circulated through the venturi by a centrifugal pump (Goulds Pump Model no: NPE 1ST1F1B4) until the desired concentration was achieved. Excess ozone was removed by a thermal-catalytic ozone destruct unit (Del Ozone). Aqueous ozone at 5 ppm or 10 ppm was tested in that study. The lower concentration (5 ppm) was applied for 5 min, and the 10-ppm ozone solution was tested for 10 min. The concentrations of ozone in solution were monitored by ozone monitor (Q450; ATI, Collegeville, PA) in both inlet and outlet lines. Moreover, the concentration of aqueous ozone in solution was confirmed by ultraviolet spectrometry method by measuring UV absorption at 258 nm (A_258_) in a spectrophotometer (Spectronic 1,201, Milton Roy Co., Houston, TX).

#### 2.6.5. Antibiofilm efficacy of the ozone-assisted CIP system

Effectiveness of the modified CIP system was assessed against unattached (planktonic) and attached (biofilm) *P. fluorescens* cell populations. Samples of planktonic cells were taken from the septum sampling port on the return line of the CIP loop (Figure 1). These samples were taken using 10-cc syringes (Becton, Dickinson & Co.) before the cleaning process starts, and after pre-rinsing, post-rinsing of alkaline cleaning, and ozone sanitization. Serial dilutions, in 0.1% peptone water, were made and 1-mL aliquots were mixed with molten TSA (Becton Dickinson & Co.) using pour-plating technique (Yousef et al., 2022). Plates were incubated at 30°C for 48 h and colonies were counted. Second type of sampling was performed to infer the effectiveness of cleaning process on the biofilm cells. The biofilm samples were taken from rough and smooth surfaces; these were stainless steel segments (Figure 2, component 12) and elbows (Figure 2, component 13), respectively. To assess the impact of CIP on *P. fluorescens* biofilm population, one segment and one elbow were removed before the whole cleaning process started, and after pre-rinsing, post-rinsing of alkaline cleaning, and ozone sanitization. Removed sections were replaced with sterile sections and the cleaning processes was resumed.

### 2.7. Biofilm enumeration

For determining the biofilm formation by the fouling setup, two small stainless-steel tubes, from the fouling loop (Figure 2, component 7), were removed each day of the 5-day biofilm medium circulation to monitor the development of robust biofilm. Each stainless tube was aseptically placed into a 50-mL screw-cap vial containing 25 mL of 0.1% peptone water. The vial was shaken manually and then the tube was rinsed with additional 10 mL of 0.1% peptone water to remove the unattached cells. Biofilm cells were removed from the inside surface of the tube by swabbing with two sterile cotton swab, the tip of which was broken off into a glass tube containing 9 mL of 0.1% peptone water. The tube, including the swab, was mixed in a vortex mixer (Fisher Scientific Industries, Inc., Bohemia, NY) for approximately 30 s. The resulting suspension was diluted in 0.1% peptone water and plated onto TSA with pour- plating method, and the plates were incubated at 30°C for 2 days (Yousef et al., 2022). The average CFU per tube was converted to CFU/ cm^2^.

For enumeration of *P. fluorescens* biofilms during the ozone-assisted CIP process, biofilm cells were removed from the inside surface of the stainless-steel segments and elbows by swabbing with three sterile cotton swabs (Fisher Scientific), the tips of which were broken off into a tube containing 25 mL of 0.1% peptone water. The tubes including the swabs were mixed in vortex mixer for approximately 30 s. The resulting suspensions were diluted in 0.1% peptone water and plated onto TSA *via* pour-plating method, and these plates were incubated at 30°C for 2 days. The average number of CFU per segment was converted to CFU/100 cm^2^.

### 2.8. Examination of biofilm by scanning electron microscopy

To examine biofilms inside the small stainless-steel tubes using SEM, some preparations were required; these preparations were executed as described previously (Speers et al., 1984; Latorre et al., 2010) with some modifications. The tubes, which were taken from the fouling system, were rinsed with 10 mL of 0.1% peptone water and then placed in glass vials. The tubes were fixed with 2.5% glutaraldehyde in 0.1 M phosphate buffer at pH 7.4 in glucose solution. The tubes were rinsed three times in 0.1 M phosphate buffer for 15 min each, and each tube was then cut carefully into halves longitudinally. The half-tube pieces were rinsed three times in 0.1 M phosphate buffer, 15 min each rinse. The washed biofilm-containing half tubes were dehydrated in increasing concentrations of ethanol (25, 50, 70, 85 and 95%) for 10 min each, and then three times in 100% ethanol for 30 min each. Dried samples were mounted onto aluminum stubs and coated with gold for 2 min in a sputter coater (Cressington, Redding, CA). The samples were imaged by SEM (Nova 400 NanoSEM, FEI, Hillsboro, OR).

### 2.9. Statistical analysis

Experiments were performed at least in duplicates and repeated independently twice. Data were represented as mean ± SD of the two independent repeats and analyzed using a statistical software (GraphPad Prism 9.0.0; GraphPad software, San Diego, CA). Analysis of variance (ANOVA), with Tukey pairwise comparison, was used to determine significant differences between treatment groups or comparing pairs of treatment. Statistical significance was considered at *p* ≤ 0.05.

## 3. Results

### 3.1. Formation of robust biofilm

**Figure 3:**
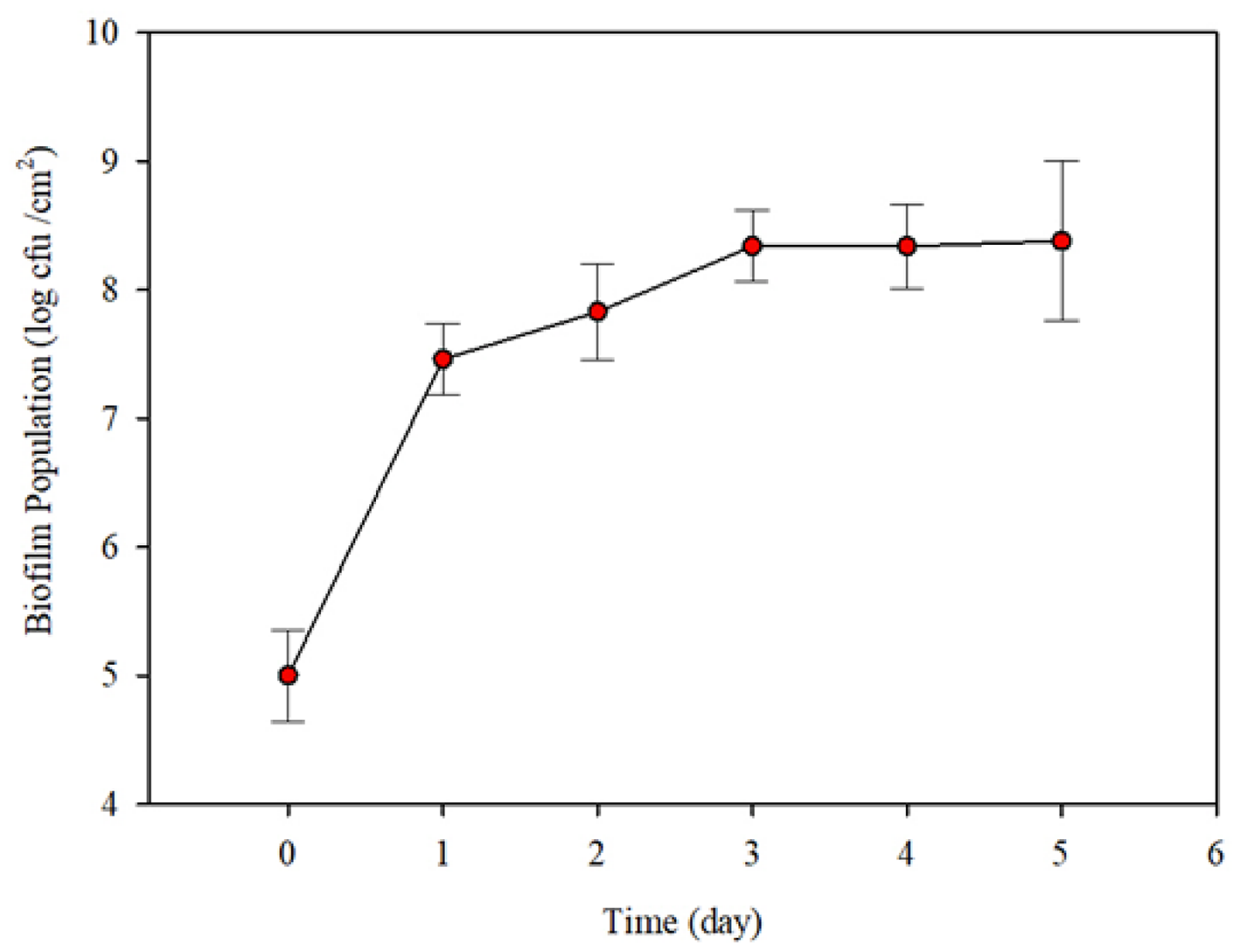
Changes in *Pseudomonas fluorescens* ATCC 25289 biofilm population formed on the interiors surfaces of small stainless-steel tubes (6.35 mm internal diameter, 19.05 mm long and internal surface area 3.78 cm^2^), after incubation in 10% diluted skim milk under stagnant conditions, followed by circulating the contents of the contamination loop at 1.5–2.0 L/h and simultaneous feeding fresh diluted skim milk at 125 mL/h. Each error bar represents ± standard deviation.

Preliminary experiments were conducted to determine the range of conditions needed for robust biofilm formation. Initial *P. fluorescens* population of biofilm cells on the surface was 1.0 × 10^5^ CFU/cm^2^ after 2 days incubation under stagnant conditions (Figure 3). When circulation by feeding fresh 10% skim milk, there was a significant increase (~ 2.46 log CFU/cm^2^) in the biofilm population count after 1 day. The population count of biofilm increased slightly up to 3 days and did not change thereafter (Figure 3).

### 3.2. Revealing biofilm structure using SEM

**Figure 4:**
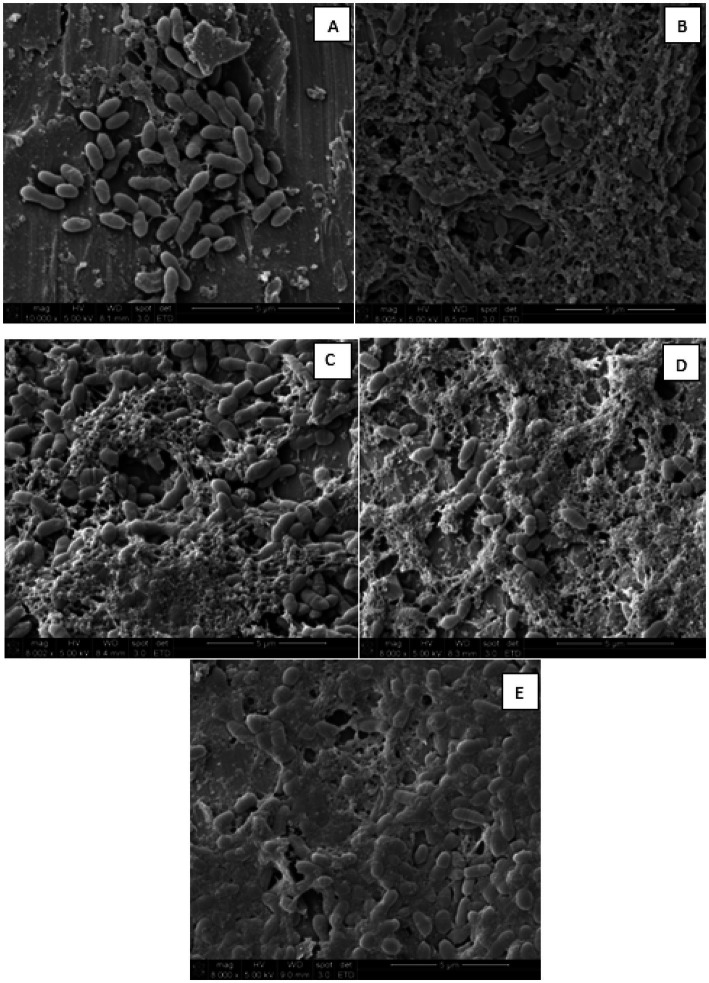
Scanning electron micrographs showing *Pseudomonas fluorescens* ATCC 25289 biofilm structure on stainless steel surface after 2-day stagnant incubation **(A)**, and after one-day circulation **(B)**, three-days circulation **(C)**, four-days circulation **(D)**, and five-days circulation **(E)** at 1.5–2.0 L/h and simultaneous feeding with 10% diluted skim milk at 125 mL/h.

The biofilm structure after 2-day incubation under stagnant conditions, and after circulating fresh 10% skim milk for up to 5 days, is shown in Figure 4. Results show that *P. fluorescens* cells were loosely attached after 2 days under stagnant incubations (Figure 4A). Without circulation and feeding fresh medium, the biofilm structure was not well-developed on the stainless-steel surface. After 1 day of circulating fresh skim milk, cells were agglomerated and covered with milk soils, and these provided the appropriate environment to allow cells growth and attachment to the stainless-steel surface (Figure 4B). On the third day of circulation, more cells were agglomerated and were surrounded by web-like strands, reminiscent of extracellular polymeric substances (EPS), with slightly more milk soil accumulation on the surface (Figure 4C). By the fourth day of circulation, the biofilm cells formed network–like structure and more EPS-like material was formed (Figure 4D). After 5 days, the biofilm cells were embedded in a compact EPS material, which is the main element in building-up biofilm on surfaces (Figure 4E). The EPS completely covered the surface of the stainless-steel tubes and, thus, the biofilm cells were permanent on surfaces and became resistant against cleaning operations. Hence, the SEM micrographs (Figure 4) support the notion that there was a continuous progress of biofilm formation even though there was no change in the count of the biofilm cells as shown in Figure 3. The longer the feeding with circulating fresh medium, the more robust was the formed biofilm.

### 3.3. Ozone-assisted CIP treatment of *Pseudomonas fluorescens* biofilm

After CIP conditions were applied, populations of planktonic and biofilm cells on rough and smooth surfaces of the processing equipment were determined in the circulating fluid, and by swabbing stainless-steel segments (rough surfaces) and stainless-steel elbows (smooth surfaces), respectively.

#### 3.3.1. Removal of 5-day biofilm from food processing equipment using ozone-assisted CIP

Based on biofilm development micrographs (Figure 4), strong biofilm buildup was accomplished after 5 days post circulation and feeding was with 10% skim milk. Hence the 5-day biofilm was developed and tested in these CIP experiments (Tables 1, 2). Initial population of the planktonic cells fed with 10% diluted skim milk for 5 days was 7.2 log CFU/mL (Table 1). For biofilm cells, 7.2–7.7 log CFU/100 cm^2^ on the smooth and rough stainless-steel surfaces, were obtained (Table 1). Pre-rinsing caused ~5.9 log decrease of planktonic cells in the fluid samples; however, the biofilm cell population decreased by ~0.92 and 1.5 log CFU/100 cm^2^ on smooth and rough surfaces, respectively. The alkaline cleaning step eliminated the planktonic cells in the fluid, and decreased biofilm cells on the smooth and rough surfaces by 2.4 and 1.4 log CFU/100 cm^2^, respectively. Ozone treatment at 5 ppm for 5 min, did not significantly decrease the number of biofilm cells on rough surface. However, there was 2.1 log CFU/100 cm^2^ reduction (*p* < 0.05) in the number of biofilm cells on the smooth surface (Table 1).

**Table 1:** *Pseudomonas fluorescens* ATCC 25289 populations recovered from the CIP system that was contaminated with 10% skim milk, inoculated with 0.5% culture, and held for 2 days, followed by circulating 10% skim milk for 5 days. The biofilm was subjected to water prerinse, alkaline cleaning (0.2% for 2 min), water post-rinse, and ozone treatment *(5 ppm for 5 min)*. Population counts were determined in fluid samples (for planktonic cells) and swab samples (for biofilm cells); the latter were taken from smooth and rough surfaces at different stages of cleaning and sanitization.

Sampling stages	Planktonic and biofilm populations* (log count ± SD)		
	Fluid log CFU/mL	Smooth surface log CFU/100 cm^2^	Rough surface log CFU/100 cm^2^
Before cleaning	7.2 ± 1.13^a^	7.2 ± 1.1^a^	7.7 ± 0.93^a^
After prerinse	1.3 ± 0.46^b^	6.3 ± 1.4^a^	6.2 ± 0.99^ab^
After alkaline cleaning	BDL^**^	3.9 ± 1.4^b^	4.8 ± 0.22^b^
After ozone treatment	BDL	1.8 ± 2.5^c^	4.3 ± 0.90^b^

^*^Different superscripts, withing the same column, indicate significant differences between cleaning stages (*p* < 0.05). ^**^Counts were below the detection level (0 log CFU/mL) of the enumeration method for fluid samples.

**Table 2:** *Pseudomonas fluorescens* ATCC 25289 populations recovered from the CIP system that was contaminated with 10% skim milk, inoculated with 0.5% culture, and held for 2 days, followed by circulating 10% skim milk for 5 days. The biofilm was subjected to water prerinse, alkaline cleaning (0.2% for 2 min), water post-rinse, and ozone treatment *(10 ppm for 10 min)*. Population counts were determined in fluid samples (for planktonic cells) and swab samples (for biofilm cells); the latter were taken from smooth and rough surfaces after different stages of cleaning and sanitization.

Sampling stage	Planktonic and biofilm populations* (log count ± SD)		
	Fluid log CFU/mL	Smooth surface log CFU/100 cm^2^	Rough surface log CFU/100 cm^2^
Before cleaning	8.4 ± 0.49^a^	7.8 ± 0.71^a^	7.8 ± 0.80^a^
After prerinse	0.3 ± 0.49^b^	6.6 ± 0.12^b^	6.6 ± 0.31^b^
After alkaline cleaning	BDL^**^	1.4 ± 2.0^c^	1.5 ± 2.2^c^
After ozone treatment	BDL	BDL	BDL

^*^Different superscripts, withing the same column, indicate significant differences between cleaning stages (*p* < 0.05). ^**^Counts were below the detection limits of the enumeration methods: 0 log CFU/mL, 0.9 log CFU/100 cm^2^, and 1.4 log CFU/100 cm^2^ for planktonic cells in the fluid, and biofilm cells on smooth and rough surfaces, respectively.

To enhance lethality of ozone against biofilm cells, ozone treatment time was extended to 10 min and the concentration was increased to 10 ppm and the biofilm inactivation results are shown in Table 2. Under these conditions, water pre-rinsing eliminated almost all planktonic cells, and decreased their count below 0.3 log CFU/mL. However, the number of biofilm cells on smooth and rough surfaces declined by only 1.2 log CFU/100 cm^2^. Using alkaline cleaning decreased the biofilm cells by ~5.2 log for smooth surfaces and ~ 5.0 log for rough surfaces. Application of ozone after the post rinse decreased biofilm cell population below enumeration method’s detection limit (0.9 and 1.4 log CFU/ 100 cm^2^) on smooth and rough surfaces, respectively, as shown in Table 2.

## 4. Discussion

*Pseudomonas* spp. are ubiquitous bacteria that readily form biofilm on food contact surfaces. Biofilm formation increases the risk of product recontamination within processing environments. This risk prompted us to develop an ozone assisted-CIP process for efficient biofilm removal. In this study, planktonic cells were less resistant to elimination by CIP treatment than were the biofilm cells (Tables 1, 2). Previous researchers found higher susceptibility of planktonic cells of different foodborne pathogens to biocides including ozone (El-Azizi et al., 2016; Fagerlund et al., 2017; Panebianco et al., 2021).

A pilot-scale ozone-assisted CIP system, which mimicked a dairy CIP process, was developed during the current study. The ozone-assisted CIP skid was connected to a processing equipment, the components of which were perceived as hard-to-clean parts (Figure 2). The most challenging step was building a robust biofilm in the pilot-scale food processing setup. The continuous feeding with the diluted skim milk medium supplied *P. fluorescens* cells with nutrients, which made them metabolically active, and hence enabled robust biofilm formation in the food processing system. Fielding et al. (2007) also observed that feeding with fresh medium during biofilm formation is necessary because of the increasing numbers of the biofilm formers which require sustained source of nutrients. In addition, the time required for forming a robust biofilm varied among studies. For example, Bremer et al. (2006) stated that 18 h were sufficient to building a biofilm in a continuous flow bioreactor and to testing the effectiveness of the cleaning process. Dufour et al. (2004) investigated different time periods (18 h, 24 h and 42 h) to develop a biofilm that is more than 5 log CFU/ cm^2^ and reported that 24 h was the most suitable time to produce the highest bacterial count in a biofilm state. In the current work, the number of cells forming the biofilm increased until the 3^rd^ day of incubation (Figure 3) after which no significant increase occurred. However, the structure of the biofilm advanced progressively until day 5, as observed by the SEM (Figure 4).

Cleaning operations were performed in this study following a procedure commonly used in industry settings. The first step, prerinsing, was carried out for soil removal from the food processing system; this effectively removed planktonic cells (Tables 1, 2). To better assess the effectiveness of the cleaning regimes, sampling of biofilm cells was implemented. The number of biofilm cells was determined by swabbing smooth (stainless-steel elbows) and rough (stainless-steel segments) surfaces. Swabbing was proved previously as an effective method for removing most biofilm cells from surfaces (Dufour et al., 2004; Redanz et al., 2021). Compared to planktonic cells, pre-rinsing decreased the count of biofilm cells by 0.9–1.5 log/100 cm^2^ only (Tables 1, 2). This limited decontamination may be attributed to the tight adherence of biofilm cells to the stainless-steel surfaces, together with the short contact time of the pre-rinsing water.

The subsequent step, which was alkaline cleaning, accomplished a great reduction of biofilm cells on smooth and rough surfaces. The alkaline cleaning is indispensable step for dairy industry’s cleaning operations because the treatment dissolves protein and fat, which are residuals of milk or milk products (Chisti, 1999). Moreover, the removal of biofilm cells from stainless steel surfaces using alkaline cleaning is attributed to (1) the peptizing action of alkaline solutions which solubilize biofilm EPS (Sharma et al., 2005; Avila-Sierra et al., 2021), and (2) the surfactant properties of the alkali which disrupt the membranes of the biofilm cells (Ammor et al., 2004). Depending on the soil load, the food industry usually uses 1–1.5% alkali cleaning solution at 70–80°C in this step to maintain cleanliness condition (Madoumier et al., 2020). However, concentration and temperature of the cleaning solution was decreased to 0.2% and 50°C, respectively, in the current study, which provides energy saving and less pollutants to environment. This approach represents substantial opportunities for efforts to reduce environmental pollution while contributing to energy savings.

Sanitization is a complementary step in CIP, and it ideally ensures complete elimination of viable biofilm cells. Without a sanitization step, CIP may not eliminate all biofilm cells (Tables 1, 2). In this study, ozone was used as the sanitizer considering it is an eco-friendly biocide, does not require heating, and demonstrates potent antimicrobial action after short contact time. Ozone could improve biofilm elimination as supported by Tachikawa et al. (2009), who reported that ozone can eliminate EPS material produced by *P. fluorescens* biofilm on glass slide. Additionally, ozone was tested as a sanitizer against several *Pseudomonas* spp., including *P*. *fragi*, *P*. *putida*, and *P*. *fluorescens*, which formed biofilms on coupons incubated in stagnant conditions for 24–72 h (Dosti et al., 2005). Considering these ozone applications were effective but at a small scale, the current study investigated the application of ozone at a pilot scale (i.e., the CIP system developed herein). When mild ozone treatment (5 ppm aqueous ozone for 5 min) was applied, biofilm population decreased significantly (*p* < 0.05) but it was not completely eliminated. The deficiency of this ozone treatment may be attributed to the recontamination originating from the unreachable points of the food processing system. Another reason could be the fact that milk was used as a feeding medium and milk contributes to the soil load and escalates curd formation; this event would decrease ozone effectiveness. Tang et al. (2010) assessed different sanitizers against biofilm formed on ultrafiltration membrane used in dairy industry. The authors found that the application of 0.5 ppm aqueous ozone for 10 min was the least effective and resulted in only 0.27 log reduction due to loss of ozone disinfection ability. However, in the current study, when strong ozone treatment (10 ppm aqueous ozone for 10 min) was implemented, biofilm cells were undetectable on equipment surfaces (Table 2). This implies that ozone application at these parameters could complement the CIP and enhance its efficacy by eliminating residual biofilm cells on food contact surfaces. Despite the strong oxidative power of ozone, no corrosion of stainless streel surfaces was noticed in the current study. However, it is likely that the use of 10 ppm ozone over extended periods causes corrosion of equipment metal surfaces. It is important that equipment manufacturers choose appropriate grade of stainless steel, which resist corrosion, in case of ozone treatment is planned (Fukuzaki et al., 2001).

## 5. Conclusion

Development of robust biofilm on a pilot-scale food processing equipment was challenging; however, overcoming this limitation enabled us to verify that ozone-assisted CIP is an efficient approach for eliminating *P. fluorescens* biofilm. This study provided evidence that standard cleaning-in-place regimes decreased *P. fluorescens* planktonic cells below enumeration method’s detection limit; however, complete elimination of biofilm cells could not be achieved. Addition of ozone as a sanitizer to the cleaning regime enabled complete removal of the biofilm cells from rough or smooth surfaces of a processing equipment. The current research implies that ozone-assisted CIP can be an efficient method for food processors to decontaminate processing equipment, particularly in the dairy industry.

## Data availability statement

The original contributions presented in the study are included in the article/supplementary material, further inquiries can be directed to the corresponding author.

## Author contributions

GS, DK, and AY designed the research and planned experiments. GS and AA analyzed the data and wrote the manuscript original draft. All authors contributed to the article and approved the submitted version.

## Funding

The study was financially supported by the Centre for Advanced Processing and Packaging Studies.

## Conflict of interest

The authors declare that the research was conducted in the absence of any commercial or financial relationships that could be construed as a potential conflict of interest.

## Publisher’s note

All claims expressed in this article are solely those of the authors and do not necessarily represent those of their affiliated organizations, or those of the publisher, the editors and the reviewers. Any product that may be evaluated in this article, or claim that may be made by its manufacturer, is not guaranteed or endorsed by the publisher.
